# Mechanisms of atrial fibrillation in athletes: what we know and what we do not know

**DOI:** 10.1007/s12471-018-1080-x

**Published:** 2018-02-06

**Authors:** E. Guasch, L. Mont, M. Sitges

**Affiliations:** Institut Clinic Cardiovascular, Hospital Clínic de Barcelona; IDIBAPS; Universitat de Barcelona; CIBERCV., 08036 Barcelona, Catalonia Spain

**Keywords:** Atrial fibrillation, Endurance, Exercise, Atrial fibrosis, Vagal tone

## Abstract

Exercise is an emerging cause of atrial fibrillation (AF) in young individuals without coexisting cardiovascular risk factors. The causes of exercise-induced atrial fibrillation remain largely unknown, and conclusions are jeopardised by apparently conflicting data. Some components of the athlete’s heart are known to be arrhythmogenic in other settings. Bradycardia, atrial dilatation and, possibly, atrial premature beats are therefore biologically plausible contributors to exercise-induced AF. Challenging findings in an animal model suggest that exercise might also prompt the development of atrial fibrosis, possibly due to cumulative minor structural damage after each exercise bout. However, there is very limited, indirect data supporting this hypothesis in athletes. Age, sex, the presence of comorbidities and cardiovascular risk factors, and genetic individual variability might serve to flag those athletes who are at the higher risk of exercise-induced AF. In this review, we will critically address current knowledge on the mechanisms of exercise-induced AF.

## Introduction

Atrial fibrillation (AF) is the most frequent sustained arrhythmia in the developed world, bearing a poor quality of life and increasing the risk of stroke and mortality. Prevalence of AF has been steadily increasing in recent years, and the number of individuals with AF is expected to double by 2060 [1]. The main factors promoting AF are ageing, structural heart disease, hypertension and diabetes, but these are absent in up to 15% of AF patients. The cause of AF in these young patients with no cardiovascular conditions has been the focus of extensive research in recent years, and obstructive sleep apnoea, obesity, tall stature and genetic predisposition have all been associated with increased risk of AF [2, 3].

**Fig. a:**
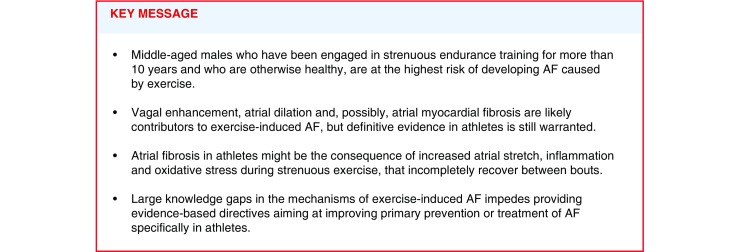
Schematic representation of the potential mechanisms underlying exercise-induced atrial fibrillation represented in a Coumel’s triangle of arrhythmogenesis, and their functional consequences

Reports published at the end of the 1990’s suggested that veteran athletes are also at a higher-than-expected risk of AF [4, 5]. Subsequent small [6] and large epidemiological studies including >1 million individuals [7] confirmed this association. Endurance training is now a well-accepted cause of AF [8]. Heavily trained athletes are, on average, at a 3–8-fold increased risk of AF [5, 6] and its prevalence is as high as 15% in veteran elite athletes [9, 10]. Intense physical activity history is reported by up to 60% of young patients with AF in the absence of any cardiopulmonary disease [2]. In the daily practice, exercise-induced AF is usually diagnosed in middle-aged males who have been practicing very intense endurance sports (e. g., marathon running [6], cycling [10], cross-country skiing [11]) in the long-term (>10 years) [12]. Not uncommonly, AF is diagnosed some years after regular training has been discontinued [6]. Overall, these data challenge the notion that the benefits of physical activity have no appreciable limits [13].

The emergence of exercise as a potential cause of AF is relatively novel, and its pathology and underlying mechanisms remain largely unknown. Few works have shed some light on the causes of exercise-induced AF, and uncertainties are still prevailing in this field. Is AF *only *a marker of extreme physical adaptation or is it associated with a pathological substrate? If so, do we have any evidence in humans of a deleterious effect of physical activity on cardiac structures? Why does the *healthy* exercise become *harmful*? Do illicit performance-enhancing substances play a role? While clinical and epidemiological evidence for exercise-induced AF is compelling, there is little evidence for a deleterious effect in the left ventricle: why do these cardiac chambers behave so differently? And, finally, why do only a few athletes develop AF? In this review, these issues will be critically reviewed on the basis of current evidence.

## Is exercise-induced AF an extreme form of physiological adaptation?

**Fig. 1:**
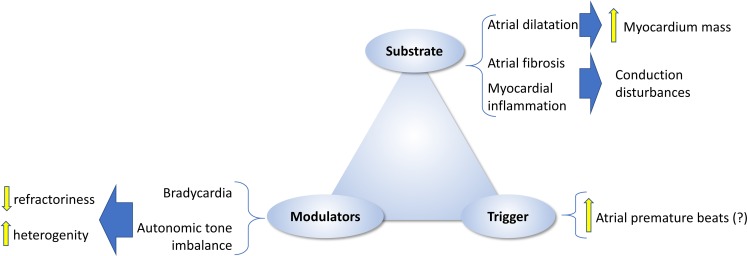
Schematic representation of the potential mechanisms underlying exercise-induced atrial fibrillation represented in a Coumel’s triangle of arrhythmogenesis, and their functional consequences

Our knowledge of the substrate that sustains AF in athletes is poor, largely speculative, based on general notions of AF pathology and, in few cases, derived from clinically relevant animal models. In the classic and simple, but useful, Coumel’s triangle, an appropriate substrate, a predisposing modulator and a timely trigger are needed in variable proportions to initiate and maintain AF (Fig. 1). The currently available evidence for the potential contribution of each of these mechanisms in exercise-induced AF is summarised in Tab. 1. Interestingly, some of the classical components of the physiologic cardiac adaptation to regular physical activity (so-called *athlete’s heart*) have also been associated to AF pathology.

**Table 1:** Evidence in animal models and in athletes supporting the potential mechanisms underlying exercise-induced atrial fibrillation

	Evidence in animal models	Evidence in humans	Main knowledge gaps
Atrial dilatation	Atrial dilatation occurs in heavily trained animals [20]	Atrial dilatation increases with exercise-load in athletes [18]	It is unclear whether atrial dilatation geometry is similar in athlete’s heart and in pathological settings
		A subgroup of athletes may be at risk of high atrial wall stress during exercise [65]	
Atrial fibrosis	Histological evidence of exercise-induced atrial fibrosis [20, 31]	Fibrosis-related biomarkers are increased in athletes [33–36]	Direct evidence of exercise-induced fibrosis in athletes is lacking
	Atrial fibrosis is central in exercise-induced AF [31]	Prolonged *p*-wave duration [18, 43]	Does increased wall stretch contributes to myocardial fibrosis pathology?
Inflammation	Local and systemic inflammation at each exercise bout [56]	Systemic exercise-load dependent inflammation has been reported [53, 54]	Local myocardial inflammation is uncertain in heavily trained athletes
Vagal enhancement & bradycardia	Reduced intrinsic heart rate [24] and vagal tone enhancement [20] have been found	Both vagal tone enhancement [18] and intrinsic heart rate reduction [25] are present in athletes	Whether parasympathetic tone contributes to AF in athletes has not been demonstrated
	Vagal enhancement governs AF inducibility [20]	Most AF episodes in athletes occur in vagal settings [4]	Could parasympathetic tone be a therapeutic target?
Atrial premature beats	Increased pulmonary vein stretch may increase arrhythmogenicity [85]	Mild [18, 29] or no [10] increase in atrial premature beats in athletes	Current evidence shows no contribution to exercise-induced AF
	No evidence for an increase in atrial premature beats/triggered activity in heavily trained animals [20, 31]		

*AF* atrial fibrillation

On the one hand, atrial size is a well-recognised independent predictor of incident AF [14]. In experimental models, dilatation of the left atrium facilitates the instauration and perpetuation of AF even in the absence of myocardial fibrosis [15]. We still do not fully understand the mechanisms behind AF promotion in dilated atria, but disparities on conduction velocity throughout the left atrium probably contribute [15]. Conduction heterogeneity in dilated atria might be originated by cellular electrophysiological changes occurring at cellular level in hypertrophied cardiomyocytes [16]. Moreover, atrial dilatation increases the atrial critical mass and facilitates the establishment of re-entrant electrical activity and AF [17]. On the other hand, atrial dilatation is a hallmark of the athlete’s heart [18]. Atrial dilatation results from the adaptation of the atria to regular training: both atria enlarge to accommodate the increased cardiac output requirements during exercise. However, the characteristics of atrial dilatation are not exhaustively known. It is currently unknown whether atrial dilation geometry differs in athletes and in patients with a heart disease. Notably, at a similar degree of atrial dilatation, atrial function seems to be preserved in athletes, but not in patients with a structural heart disease [19].

A slow heart rate is a common finding in well-trained individuals. For decades, bradycardia in athletes has been attributed to an imbalance in autonomic tone characterised by parasympathetic tone enhancement and sympathetic tone withdrawal. Parasympathetic tone shortens the atrial refractory period and thereby facilitates re-entry and AF. Results in an animal model suggest that exercise enhances parasympathetic tone partially through an increased cardiac sensitivity to acetylcholine, an effect mediated by downregulation of regulators of G protein signalling (RGS) [20]. In this model, parasympathetic enhancement was central in the early stages of exercise-induced AF pathology [20]. Notably, most AF relapses in athletes occur in vagally-dominant situations such as during sleep or after meals [4]. Remarkable clinical implications may derive if parasympathetic tone is confirmed as a main driver of exercise-induced AF. For example, antiarrhythmic drugs with vagolytic properties (e. g., dysopiramide [8, 21]) could be favoured over other options, and intracardiac autonomic ganglia could become a primary target in ablation procedures [22]. Conversely, adrenergic-mediated AF is less frequent in athletes [4]. Although the parasympathetic and sympathetic tone shorten atrial refractoriness to a similar extent, the more heterogeneous atrial parasympathetic innervation yields a larger arrhythmic susceptibility [23].

The notion that parasympathetic tone enhancement is the sole cause of bradycardia in athletes has recently been disputed. D’Souza et al. elegantly demonstrated in mice that a reduction in intrinsic heart rate (i. e., changes in the sinus node function independent of autonomic regulation) through HCN4 downregulation governs bradycardia in trained individuals [24], thereby supporting conclusions from previous studies in athletes [25]. Moreover, the modification of the intrinsic properties of the sinus node is consistent with reports pointing to a high prevalence of sinus node disease and pacemaker requirement in athletes [10]. The increased atrial refractoriness dispersion during bradycardia might indeed link intrinsic heart rate reduction to exercise-induced AF pathology [26].

It is likely that both autonomic tone-mediated remodelling and intrinsic heart rate-mediated remodelling contribute to bradycardia in athletes, and that the balance between both factors change with training intensity and/or sport discipline [27]. In the general population, either parasympathetic tone-driven bradycardia or intrinsic heart rate-driven, bradycardia is associated with a higher risk of AF [28].

In the general population, atrial premature beats may trigger AF events in the presence of other predisposing factors or, when very frequent, may be the main etiological factor. It has been postulated that endurance athletes present with a higher burden of atrial premature beats than sedentary individuals [18, 29]. Nevertheless, it remains unknown whether such a mild increase is enough to significantly contribute to the AF burden in athletes.

Both atrial enlargement and bradycardia are more evident in endurance sports athletes than in strength sports (e. g., weight-lifting) practitioners. In parallel, exercise-induced AF is far more frequent in endurance athletes. On this basis, some authors hypothesised that AF might be an extreme manifestation of the physiological athlete’s heart. However, recent results from our and other groups disputed this notion. Works in animal models suggested that atrial fibrosis, a clearly pathological hallmark, could contribute to exercise-induced AF pathology. Atrial fibrosis disrupts normal electrical conduction in the atrium and, possibly, interferes with myocyte-fibroblast electrical coupling, thereby facilitating the establishment of re-entries and, eventually, AF [30]. In a rat model, we first found that a 16-week intense training protocol increased AF inducibility in an electrophysiological test. In addition to atrial dilatation and an enhanced parasympathetic tone, we observed increased atrial interstitial fibrosis [20], a finding that has been subsequently confirmed by others [31]. Of note, both studies found a modest (≈60%) increase in atrial fibrosis [20, 31], contrasting to much larger atrial fibrosis deposits in other pathologic settings known to be associated with AF, such as left ventricular dysfunction or valve disease. In these conditions, atrial fibrosis increases up to 500% [32]. Although some experimental work suggests that exercise-induced atrial fibrosis drives AF inducibility [31], it is also plausible that additional contributing factors should be present, at least in the early stages of exercise-induced AF.

## Do we have data on pathological remodelling in humans?

The confirmation in humans of a pathologic substrate in intensively trained individuals is still pending. In particular, the need of invasive tests to assess atrial fibrosis hampers its confirmation in athletes. Atrial biopsies are an unrealistic approach to quantify myocardial fibrosis in apparently healthy individuals, and magnetic resonance techniques are still underdeveloped. Therefore, only indirect estimates are currently available.

Results on plasmatic biomarkers are consistent with the presence of a profibrotic state in some trained individuals. Active or veteran athletes present with higher levels of pro-fibrotic markers, such as galectin-3 [33], ST2 [34], certain circulating pro-fibrotic microRNAs such as mir-21 [35], and collagen turnover peptides PICP, CITP and TIMP-1 [36]. These plasmatic biomarkers have been associated with incident or recurrent AF in clinical works in the general population and in patients with structural heart disease [37–40]. It should be noted, though, that the interpretation of these results is complex and, at best, suggest the existence of a pro-fibrotic systemic environment that could favour atrial fibrosis.

Echocardiographic parameters such as atrial strain have been used as surrogate markers for the degree of atrial fibrosis in patients undergoing mitral valve surgery [41]. However, results in well-trained endurance athletes are conflicting [42, 43].

The ECG is an easy, widely available tool that may be useful to provide a rough approach to atrial structures. In patients with mitral valve disease, *p*-wave duration associates with atrial fibrosis and size [44, 45], and flags those individuals at an increased risk of AF. Endurance athletes present with an accumulated physical activity-dependent *p*-wave prolongation [43, 46]. In football players, such a prolongation is independent of atrial size, and thereby fibrosis evolves as a plausible underlying substrate [47].

## What triggers pathological remodelling?

Secondary prevention trials [48] and observational studies assessing incident AF in older individuals or in individuals with a high burden of cardiovascular risk factors [49–51] demonstrate that low to moderate load of physical activity is safe and may even be antiarrhythmic. Conversely, a high exercise load may promote AF in some individuals. Overall, these findings depict a U-shaped relationship between exercise load and AF incidence [2, 12]. It is unclear which are the determinants promoting the transition from safe exercise to the appearance of exercise-induced atrial fibrillation. Chances are that atrial dilatation or bradycardia beyond a certain level in well-trained athletes facilitate AF. It is also possible, though speculative, that an increase in AF incidence associates with the establishment of atrial fibrosis.

**Fig. 2:**
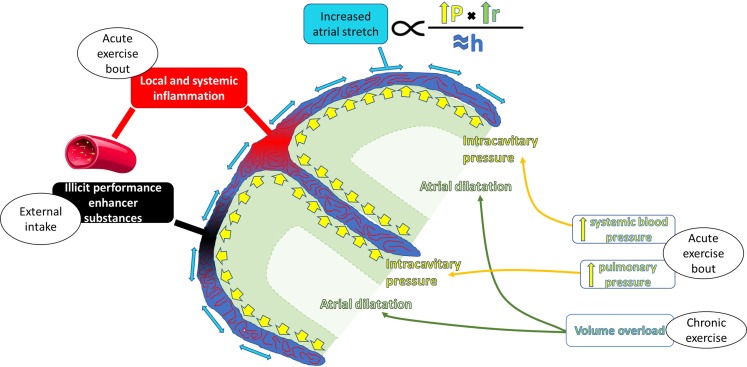
Potential factors leading to a pathological atrial remodelling in athletes (atrial fibrosis). Systemic blood pressure and, particularly, pulmonary pressure promote an increase in atrial intracavitary pressure during exercise. In the presence of chronically dilated atria and limited ability to increase wall thickness, atrial wall stretch has remarkably increased, which may promote the activation of profibrotic mechanisms. A pro-inflammatory status during each exercise bout and intake of an illicit performance enhancer may also contribute. *RA* right atrium, *LA* left atrium

In this regard, the mechanisms behind the potential instauration of atrial fibrosis remain unknown. Fig. 2 summarises the factors that have been postulated to contribute to exercise-induced atrial fibrosis. It is possible that systemic or mechanic insults during strenuous exercise bouts inflict cumulative microstructural myocardial damage. As for the right ventricle, it has been postulated that such damage develops after incomplete recovery between exercise bouts, thereby leading to permanent damage to the atria.

Pro-inflammatory insults and low-level inflammation have been associated with AF incidence in the general population [52]. Although regular physical activity has been demonstrated to yield a systemic chronic anti-inflammatory effect, each exercise bout disturbs the inflammatory balance and prompts a pro-inflammatory status. Intense exercise transiently increases neutrophil count and induces the release of pro-inflammatory cytokines such as interleukin-6, interleukin-8, C‑reactive protein and ST2 [34, 53–55]. Systemic inflammation may locally extend to the myocardium. In a swimming-based animal model, extenuating exercise bouts were associated with myocardial leukocyte infiltration [56]. Interestingly, local inflammation mediated by stretch-activated tumour necrosis-alpha (TNF-α) seems to be critical in exercise-induced atrial fibrosis pathology [31]. In humans, a transient p‑wave prolongation after ultra-distance races was observed independently of atrial size, leading the authors to postulate that transient inflammatory infiltration or oedema could cause such conduction disturbances [57]. Changes in plasma of TNF-α and interleukin-12p70 concentrations correlate to right ventricular dysfunction after ultra-distance races in well-trained athletes [54]. Oxidative stress, which has also been linked to an increased AF incidence [58], increases in a load-dependent way after exercise [59]. Several attempts have been undertaken to blunt the pro-inflammatory and oxidative status after strenuous exercise with nutritional supplements and drugs, yielding conflicting results on the systemic inflammatory status [60, 61], and showing no benefits on cardiac haemodynamic overload markers [62].

A transient increase of cardiac necrosis markers (e. g., troponin I and troponin T) after long distance races was reported some years ago and claimed to be a marker of cardiac necrosis during exercise. Plasmatic troponin levels were associated with right, but not left, ventricular transient dysfunction after strenuous exercise [63], but a worse outcome in those athletes with repetitive troponin elevation has not been demonstrated. However, our current understanding of troponin release during long races involves a process of cardiomyocyte membrane permeabilisation rather than pathological ischaemia [64]. To date, repetitive myocardial ischaemia and necrosis cannot be established as a source of AF substrate.

Each exercise bout involves a volume overload that superimposes a mechanical stress to which the thin atrial wall is particularly sensitive. Wall stress positively correlates with intracavitary pressure and size, and inversely with wall thickness. Atrial enlargement implies that wall stress is higher, according to Laplace’s law (Fig. 2). Additionally, if the curvature of the atria changes (i. e., becoming more elliptic), wall stress might also increase (the flatter the wall, the higher the wall stress) [65]. Atrial natriuretic peptide (ANP) levels, a marker of atrial stretch, is increased at rest in athletes in comparison with healthy individuals [66, 67]. Atrial pressure increases during exercise [31, 68], further increasing wall stretch and prompting a subsequent deleterious remodelling. Such deleterious effects might be particularly notorious in a subset of athletes with dilated and dysfunctional atria [69]. Increasing loads of physical activity are associated with an acute, dose-dependent transient atrial dysfunction, which becomes severe after very intense and prolonged bouts [70]. As aforementioned, increased atrial wall stress has been shown to trigger TNF-mediated activation of local inflammation, eventually leading to atrial fibrosis in an animal model [31]. Blocking TNF-α did prevent exercise-induced atrial fibrosis and inducibility. Speculatively, hidden hypertension or hypertension during exercise bouts may further exacerbate this increase in atrial wall stress and accelerate it [71]. Overall, increased atrial wall stretch appears as an attractive trigger for pathological remodelling after strenuous exercise, but definitive proof remains elusive.

## What makes the atrium different, why is it selectively affected?

For a long time, research in sports cardiology focussed on the study of left ventricle adaptation to variable amounts of physical activity. It was convincingly demonstrated that left ventricle adaptation to high loads of physical activity does not usually convey pathological stigmas. Proteomic studies emphasised the physiological remodelling of the left ventricle in intensively-trained animals [72], and some studies suggest that physical activity may even protect against ventricular arrhythmias [73, 74]. However, the atria and right ventricle were scarcely studied.

The recent demonstration of potentially deleterious effects of very high doses of physical activity has moved the focus towards the atria and the right ventricle [12]. The physiological remodelling of the left ventricle contrasts with the identification of atrial and right ventricular arrhythmias in athletes. Morphological, functional and molecular differences between left ventricle, right ventricle and both atria underlie a distinct response to high levels of physical activity [75].

As described in the previous section, the morphology of the atrium makes it particularly vulnerable to haemodynamic disturbances. Haemodynamic overload promotes atrial dilatation and, subsequently, increases wall stretch. Conversely, the ability of the left ventricle to respond to repetitive mechanical overload by thickening its myocardial walls enables the left ventricle to maintain wall stretch within a non-deleterious range [75, 76]. This adds to differences in the cellular and molecular characterisation of atria and ventricles. Indeed, atrial fibroblasts show an enhanced reactivity to pathological stimuli in comparison with ventricular fibroblasts [77], resulting in a remarkably larger atrial than ventricular fibrosis burden upon the instauration of non-ischaemic heart failure in animal models [78].

Altogether, clinical outcomes, morphological characteristics and fibroblast reactivity data suggest that the atria have a greater sensitivity to the haemodynamic overload than the left ventricle, potentially justifying that the atria are primarily affected by the deleterious consequences of strenuous physical activity [12].

## Are performance-enhancing drugs a plausible explanation for exercise-induced AF?

Performance-enhancing drugs have been postulated to contribute to AF burden in athletes. Nevertheless, the obscure nature of doping hinders any robust conclusion and thereby this issue remains, at best, speculative. Available data from athletes who have been banned from competition suggest that the most commonly used substances in high-level endurance-trained athletes who aim to improve their performance are erythropoietin (EPO), anabolic-androgenic steroids (AAS), and stimulant and sympathomimetic drugs.

Erythropoietin and its derivatives increase erythrocyte synthesis and oxygen supply to peripheral muscle, evolving as a tempting drug for endurance athletes. To date, however, there is no data reporting AF as a significant side effect of EPO.

The use of AAS has been associated with AF in isolated case reports [79]. A study with body-builders recently demonstrated that chronic anabolic steroid administration associates with a prolonged atrial electromechanical delay [80]. Atrial electromechanical delay predicts new-onset AF, likely reflecting underlying substrate abnormalities [81].

Sympathomimetic drugs, such as ephedrine and amphetamine derivates, are used as stimulants and might trigger AF; a specific form of apoptosis in myocardial biopsies termed eosinophilic bands has been associated with ephedrine intake [82].

Nevertheless, we should note that the use of performance-enhancing substances is not exclusive for long-distance endurance sports. Rather, their use is also relatively common in bodybuilders and weight-lifters, although no reports demonstrating an increased risk of AF in these cohorts has been published. It should be acknowledged, though, that it is biologically plausible that the effect of performance-enhancing substances is boosted in those endurance athletes in whom both a larger chamber dilatation/fibrosis and parasympathetic tone enhancement occur.

## Why do only few athletes develop AF?

**Fig. 3:**
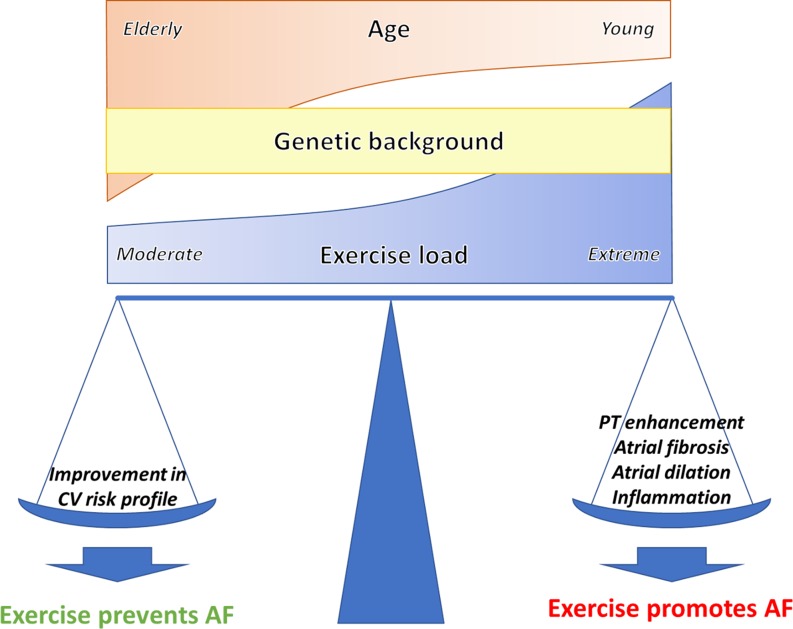
Representation of the factors contributing to the balance between the antiarrhythmic and the pro-arrhythmic effect of exercise

While exercise is performed by a large part of the population, only some athletes will develop AF. Moreover, some individuals may get protected by exercise from AF, as aforementioned [48–51]. Fig. 3 summarises the potential factors involved in this complex relationship. In terms of AF incidence, the arrhythmogenicity of exercise likely results from the net balance between its *beneficial* (e. g., improvement of risk factors burden) and its potentially pro-arrhythmic (e. g., atrial fibrosis) effects; age and the presence of cardiovascular risk factors modulate this relationship [12]. Elderly individuals accruing several cardiovascular risk factors may benefit from exercise [51]. Conversely, middle-aged individuals without cardiovascular risk factors may be more prone to exercise-induced AF [7].

On top of these factors, it is evident that some degree of genetically-derived interindividual variability facilitates the development of a pathological remodelling or enhance athlete’s heart features [69]. Men are apparently at a higher risk, likely because of bigger atria and a more extensive remodelling as compared with women [42]. Nevertheless, longer follow-up in contemporaneous cohorts of women is warranted [83]. The presence of certain genetic mutations or polymorphisms may put some athletes at a higher risk. A mutation in a subunit of the IK_s_ potassium channel has been shown to confer an increased sensitivity to atrial stretch and could therefore facilitate AF during hypertension or in athletes [84]. Unfortunately, to date, there are insufficient data that reliably identify those athletes who are at risk of exercise-induced AF.

## Conclusions

The higher incidence of AF in athletes is now well-accepted, but its causes remain elusive. Atrial dilatation and parasympathetic enhancement are likely contributors. Some data in animals suggest that extreme physical activity associates with a pathological remodelling involving atrial fibrosis. Transient inflammation and an increase in atrial wall stress, associated with uncomplete recovery between exercise bouts, could trigger the development of atrial fibrosis. Nevertheless, confirmation in humans is still waiting, largely due to limitations in registries and histological confirmation. On the other hand, the contribution of performance-enhancing substances does not appear to be remarkable. Overall, data are still insufficient to adopt specific prevention and diagnostic or prognostic strategies in the clinical setting. With our current knowledge, the potential risk of AF should not be used to limit the amount of physical activity.
